# Determining professional nurses’ knowledge on the performance management and development system in Tshwane

**DOI:** 10.4102/curationis.v38i1.1186

**Published:** 2015-06-05

**Authors:** Moselene A.R. du-Plessis

**Affiliations:** 1 Department of Nursing Science, University of Pretoria, South Africa

## Abstract

**Background:** The implementation of the Performance Management and Development System (PMDS) is seen as being vital to the quality of care, resource management and inter-professional work. However, there is evidence that, at present, professional nurses lack the knowledge to implement such a system.

**Objectives:** The aim of this study was to explore and describe professional nurses’ knowledge regarding the implementation of the PMDS in selected primary healthcare clinics in Tshwane.

**Method:** A quantitative, descriptive, exploratory design was used and convenience sampling was used to recruit respondents. A self-administered structured questionnaire was used to collect data from 39 professional nurses who were involved in managing the work performance of employees. The data were analysed quantitatively with Statistical Analysis System version 8.2 software.

**Results:** The respondents had limited knowledge about the implementation of the performance management and development system.

**Conclusion:** There is a need for educational initiatives before, during and after the implementation of the PMDS, such as quarterly PMDS workshops and routine catch-up training. These would address the lack of knowledge of respondents as seen from the results.

## Introduction

The Performance Management and Development System (PMDS) has emerged as a key fundamental and comprehensive tool for the management and measurement of employees’ work performance in the public health sector (Paile 2012:2). The PMDS is a cyclical process in which employees and managers collaborate in setting standards for their expectations, giving and receiving feedback, reviewing results and providing rewards based on performance results. According to Adejoka and Bayat (2014:9), the PMDS serves as a support tool that enables managers to better facilitate the work of the people reporting to them. It is a process by which agreement is reached on where employees should be heading, thus ensuring that they stay on course.

## Background

Quality service delivery is becoming increasingly difficult as a result of the global shortage of nurses, especially professional nurses, available to render high-quality nursing services (Stavrianopoulos 2012:393). During 2008, an average of 40.3% of professional nurses’ posts were vacant throughout South Africa:

The provinces with the highest percentages of vacancies were the Eastern Cape (53.6%), the Free State (51.6%), Limpopo (43.7%), Kwa-Zulu Natal (39.6%) and Gauteng (34.4%). These percentages of vacant professional nurses’ posts indicate a dire necessity to retain the currently professionally active nurses. (Mokoka, Oosthuizen & Ehlers 2010:3)

For these reasons, it is essential that measures be introduced to assist the limited number of nurses of all categories, in such a way that they can practise nursing both safely and effectively. The PMDS ‘has been one such effort to achieve better patient outcomes, better system performance and better professional development (Batalden & Davidoff 2007:2, as cited in Millar 2013:1). ‘Rather than effort alone, it is based on THE improvement of systems and processes’ (Berwick 1996, as cited in Millar 2013:1).

The PMDS was introduced in April, 2001 within the South African healthcare system as a key measure to ensure that human resource strategies are used to support the organisation, providing a means for assessment and improvement of the performance of both individual and institutional against predefined strategies and objectives (Adejoka & Bayat 2014:9).

The purpose of the PMDS is aimed at making a uniform performance management and development system available to the public healthcare sector. The PMDS also ensure that employees are equipped with the necessary skills, knowledge and competencies in order to respond to the ever-changing roles and activities within the institution. In addition, the PMDS aims to affirm that performance is managed on a consistent basis so as to ensure that objectives are obtained by reviewing past performance, assessing current performance, setting performance objectives, improving current performance and assisting in career development planning (Letsoale 2007:6)

### Literature review

The content of the PMDS documentation addresses role-based competency expectation for each employee, regardless of job description. As a result, the employee can then determine how these competencies translate into work performance in specific jobs (Marquis & Huston 2009:586). The PMDS is based on a balanced scorecard that measures the employee's work performance levels (1 = unsatisfactory to 5 = outstanding). Hough *et al.* (2008:194) maintain that the balanced scorecard, as a measure of performance, clearly communicates the links between organisational inputs (human and physical), processes (activities, interventions) and outcomes, focusing on the importance of managing these components to achieve the organisational strategic objectives. Muthwa (2007:32) believes that the accuracy of data collection throughout the PMDS cycle is fundamental, because the information gathered guides decisions made to improve the quality of service, whereas decisions made on poor or inaccurate data both compromise and delay service delivery.

### Performance planning

Performance planning means that each employee will set individual goals and measures that are linked directly to the strategic objectives of the organisation. The process of developing individual goals and measures starts by cascading the organisation's strategic objectives down through the departments to the individual employee. Once the manager of each department has set departmental goals and objectives, each person in the department should be assisted by means of joint goal-setting sessions, to set his or her goals and objectives.

### Performance agreement

The performance agreement (PA) is the cornerstone of performance management at the individual level. All employees are obliged to enter into and sign PAs for the new PMDS cycle by 30 April each year (Muthwa 2007:32). According to Kgomotso (2011:16–17), the criteria for assessment of an employee's work performance, contained in the PA, consist of key performance areas (KPAs) and generic assessment factors. By entering and signing the PA contract, the employees implicitly guarantee that they are capable of doing the work and that they undertake to perform their tasks competently and without negligence, at the agreed level.

### Performance monitoring

Performance monitoring at the individual level needs to be maintained continuously. This ensures that predetermined service delivery standards are met, allowing for improved planning, facilitating reporting, predicting possible failure and determining the kind of support that may be required in order to strengthen organisational capacity (Marriner-Tomey 2004:406). According to Letsoalo (2007:18–20), the only way for employees to grow realistically and to develop in their work is through monitoring and constructive feedback. Employees need to hear how they are doing – the positive and the negative – on a regular basis. If employees only hear something about their work performance once a year, they are being left in the dark about how they are doing on a daily basis.

### Performance review

Performance review forms an integral part of the monitoring process throughout the entire PMDS cycle. The core purpose of a performance review session is to initiate dialogue that will help the employees to improve their performance. The more actively employees participate in the discussions, the more likely it is that the root causes and obstacles to optimal performance will be uncovered and constructive ideas for improvement will be raised (Department of Local Government and Traditional Affairs 2007:17; Kirkpatrick 2006:166).

### Performance assessment

The performance assessment at the end of the PMDS cycle remains a prominent feature. According to Nel *et al.* (2005:318), direct supervisors or managers play a vital role in the assessment process, as they are responsible for the management and assessment of the performance activities of subordinates directly under their jurisdiction. For this reason, knowing to which extent nurses are performing as required is an important factor in the rendering of quality service; and information gained from the PMDS is therefore crucial for individual and organisational viability.

### Aims of the study

The purpose of this study was to determine and describe the knowledge of professional nurses regarding the implementation of the PMDS in selected Primary Health Care (PHC) clinics within the Tshwane district of Gauteng.

The objective of this study was to determine and describe professional nurses` knowledge regarding the implementation of the PMDS in relation to PA and planning, performance monitoring, performance review and perfor­mance assessment in selected PHC clinics within the Tshwane district of Gauteng.

### Definition of key concepts

The following concepts have been clarified for this article:

#### Performance management and development system

The PMDS is ‘… a system [*that*] describes the performance management cycle and processes of the organisation related to performance planning, monitoring, measure­ment, review, and reporting, in accordance with the organisation's strategic plan’ (Muller, Bezuidenhout & Jooste 2008:100–101).

#### Nurses’ knowledge

Nurses’ knowledge is the awareness and understanding of facts, truth or information gained through practical and theoretical experiences that are needed to care for patients (Hall 2005:36). In the context of the study being reported here, ‘knowledge’ refers to the ability to understand and apply basic facts to changing situations.

#### Professional nurse

A professional nurse is:

[*a*] person who is qualified and competent to independently practise comprehensive nursing in the manner and to the level prescribed and who is capable of assuming responsibility and accountability for such practice. (South Africa 2005 – *Nursing Act* 33 of 2005:s. 30[1])

#### Problem statement

Professional nurses are the key role-players in the implementation of the PMDS. Despite having the PMDS in place since 2001, professional nurses at PHC clinics in the Tshwane District of the Gauteng Province verbalised that they find the PMDS to be time-consuming, confusing, non-beneficial and that it does not drive performance. It is thus clear that it cannot be assumed that everyone involved in the implementation of the PMDS knows how it works and what the benefits are.

#### Research design

A quantitative design was applied, with an explorative and descriptive approach. The chosen approach enabled the author to describe the knowledge of professional nurses regarding the implementation of the PMDS working at the two selected PHC clinics.

### Research approach and method

A non-experimental descriptive survey making use of a questionnaire was carried out to collect data from professional nurses in two PHC clinics in Tshwane district in Gauteng during the first week in April 2010. Brink, van der Walt and van Rensburg (2006:148) refer to a questionnaire as a self-report instrument where the respondents write their responses to printed questions on a document. Existing instruments were not suitable or relevant to nurses in the context of this study. A 23-item multiple choice questionnaire consisting of two sections was designed by the author from literature consulted and with the help of a professional statistician, academics in nursing and specialists in the field. This approach was necessary in order to obtain the objective of this study, which was to determine and describe professional nurses’ knowledge regarding the implementation of the PMDS.

#### Data collection instrument

Section A of the questionnaire consisted of six questions regarding demographic data: gender, age, nursing qualifications, job title, years of experience and previous PMDS workshop attended. Section B comprised 17 knowledge-related questions aimed at determining the knowledge respondents hold regarding the implementation of the PMDS. Each knowledge-related question was based on concepts of implementation of the PMDS: performance planning, PA, performance monitoring, performance review and performance assessment. Respondents were requested to choose any one out of four options to measure their opinions in relation to the given statement. A total of 23 response items were assessed.

#### Population and sampling

According to LoBiondo-Wood and Harber (2010:226), convenience sampling is a non-probability method used to select participants who are readily assessable to the researcher. Convenience sampling for this study was deemed to be most suitable because the participants had experience and were familiar with the implementation of the PMDS.

The sample population for this study consisted of 60 professional nurses working at two selected PHC clinics in the Tshwane District of the Gauteng Province. A total number of 32 professional nurses worked in the first selected clinic and 28 in the second selected PHC clinic. Six professional nurses, three from each clinic, participated voluntarily in the pre-testing of the questionnaire and were excluded from the main study. The remaining 54 professional nurses participated in the main study. Only 39 professional nurses completed the questionnaire at the time of data collection. They were all selected conveniently on the basis that they were familiar with the implementation of the PMDS.

#### Data collection method

The researcher obtained permission from the facility managers to address the professional nurses at pre-arranged times and dates in the seminar rooms at each of the clinics. During these visits, the objective and purpose of the study were explained, after which they were invited to participate. The researcher distributed and collected the completed questionnaires at both clinics on the same day. The same sealed box used to deposit the questionnaires after completion was used for both clinics. Of the 54 questionnaires that were handed out, 39 (72%) were returned.

#### Data treatment

The purpose of data analysis is to categorise, order, manipulate and organise raw data so that the information derived from such data can be described in meaningful terms (Brink *et al.* 2006:170).

Descriptive statistics that sought to describe the frequencies and percentages were used for analysis of data.The Statistical Analysis System (SAS) version 8.2 software program (SAS Institute Inc., Cary, NC 1999) was used to analyse the data.

## Ethical considerations

In accordance with ethical guidelines (Bless, Higson-Smith & Kagee 2006:142–145), permission to conduct the research was obtained from the Faculty of Health Sciences Research Ethics Committee of the University of Pretoria, the Regional Director of Health: Tshwane/Metsweding district, and the facility managers of the two selected clinics. The clearance number for this study was S21/2008. The research procedure was explained and respondents were given a chance to read through the covering letter in order for verbal and written consent from them to be obtained. Respondents were informed about their right to withdraw from the study at any given time. They all participated voluntarily without being offered any incentives for participating in the study.

Respondents were reassured of anonymity and confidentiality. No personal identification was required and respondents were requested to refrain from writing their names or signatures on the questionnaires. The anonymity of the PHC clinics was also protected by making it impossible to link specific information to the specific PHC clinics, because the same box was used to collect all the completed questionnaires from both clinics.

## Trustworthiness

### Measures to ensure validity and reliability

Content validity in this study was grounded on the extent to which the questions in the questionnaire and the ratings obtained from the respondents are typical of the possible questions that a researcher could ask about the concepts being measured (Leedy & Ormrod 2000:92). The questions were formulated in view of existing literature.

The questionnaire was pretested to validate its practicability and appropriateness. The researcher used convenience sampling as a method to recruit six professional nurses working at the two selected PHC clinics – three professional nurses from each clinic. They all participated voluntarily in the testing of the questionnaire and were excluded from the main study. Content validity was further ensured by a statistician who coded the questionnaire and experienced specialist in the field nursing and academic research who assessed the questionnaire.

## Results and discussion

### Section A: Demographic profile of participants

The demographic profile of the participants illustrates essential and relevant information pertaining to the respondents’ biographical data. Nursing is generally seen as being a female-dominated profession and the findings of this study corroborated this, with 37 (95%) female participants and two (5%) men.

Twenty-three of the respondents (59%) were between 25 and 39 years of age, as shown in Table 1. The ages of the respondents were indicative of their familiarisation with a very structured form of performance appraisal such as the PMDS.

**TABLE 1:** Ages of respondents (*N* = 39).

Age	Frequency
25–39 years	23 (59%)
40–59 years	14 (36%)
60 years and above	2 (5%)

The older staff members would most likely not have been exposed to such a comprehensive system, as performance appraisals prior to 2001 did not make provision for the involvement of subordinates in the whole process.

**FIGURE 1:**
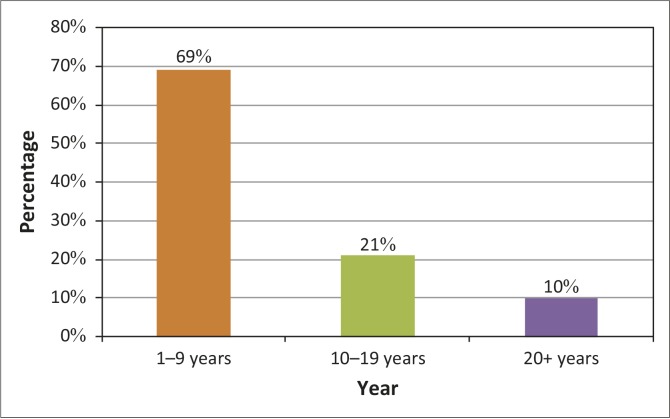
Respondents’ years of experience in the primary healthcare services (*N* = 39).

Figure 1 shows that almost a third (*n* = 27; 69%) of respondents have been working in a PHC for less than 10 years and 29 (74%) held a qualification in community nursing.

The PHC approach became the focus of the health service delivery after 1994 (20 years ago). The time participants had spent PHC could have contributed to aligning all efforts toward the achievement of their goals. As the vast majority (*n* = 29; 74%) of respondents have a qualification in community nursing; they should have been well acquainted with what needed to be achieved in the clinics. The PMDS determines the extent to which the PHC goals are achieved.

### Section B: Knowledge of the respondents regarding the system

#### Workshop attended by the participants

Twenty-one (55%) of the respondents had never attended any PMDS workshop, whilst 18 (45%) had. According to Kondrasuk (2011:65–66), great emphasis should be placed on the training of those involved in PMDS. Through training programmes, one becomes conversant with important legislation, objectives and other major aspects in relation to the PMDS. The low percentage of PMDS workshop attendance could contribute to the feeling on the part of professional nurses that the PMDS was time-consuming, confusing, non-beneficial and does not drive work performance.

#### Performance management and development system

Analysis of the results revealed that 25 (64%) of the respondents did not know who was responsible for the implementation of the PMDS and 22 (56%) had no idea about the key principles that govern the PMDS.

**FIGURE 2:**
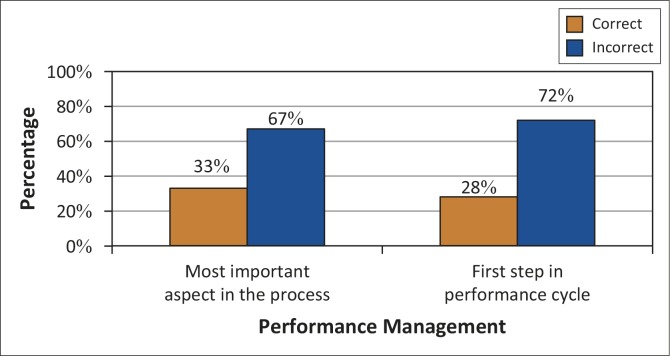
Knowledge regarding the most important aspect of performance management and the first step in the cycle (*N* = 39).

With regard to identifying the most important aspect of the PMDS process, 26 respondents (67%) lacked knowledge thereof and 28 (72%) answered the question about the first step in the performance cycle incorrectly. Because of this lack of knowledge, employees might not be clear about which goals they are expected to achieve (Figure 2).

#### Performance planning and agreement

**FIGURE 3:**
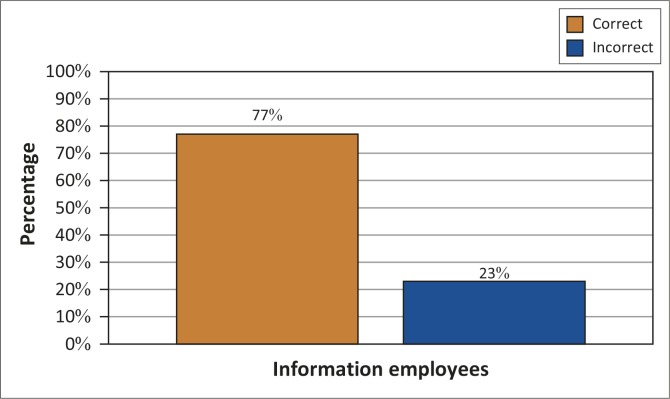
Knowledge about the information employees obtain from the key performance areas in the work plan (*N* = 39).

Of concern is that 24 (62%) of the respondents lacked knowledge pertaining to the date for signing a PA and 30 (77%) were unaware that the work plan contained the essence of the performance agreement when entering into said agreement. Before signing a PA, the employee should be clear about what should happen and when. If this does not happen, planning and future work performance of the individual employee will be compromised, which has a direct influence on the quality of care. It is thus important that those involved in the using of the system know how it works (Figure 3).

The findings of this study revealed that 30 (77%) of the respondents had adequate knowledge regarding what the KPAs indicated to the employee and how the performance will be measured. This would therefore imply that the majority of participants were knowledgeable about the role of the KPAs.

#### Performance monitoring

**FIGURE 4:**
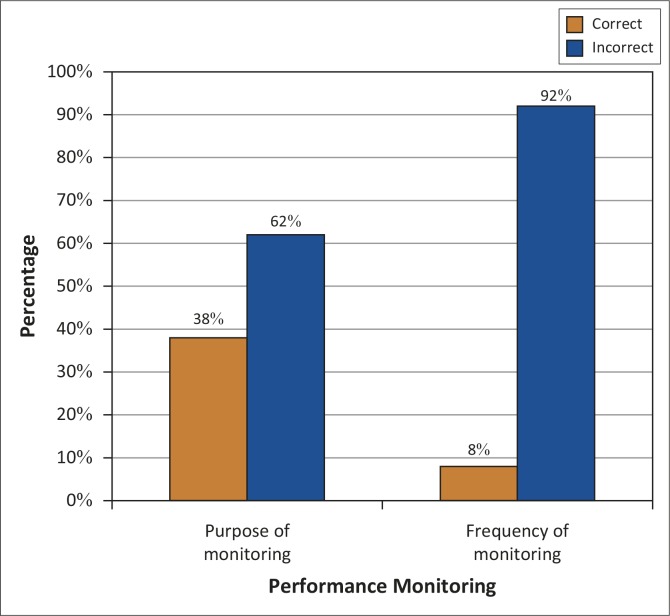
Knowledge regarding performance monitoring (*N* = 39).

The vast majority of respondents (92%; *n* = 36) did not know how often monitoring of the employees should take place and 24 (62%) lacked knowledge regarding the purpose of monitoring. In nursing, the concept of monitoring is a well-understood function. The fact that respondents could not correctly answer the question reflected negatively on the quality of the nursing care they are rendering. This lack of knowledge could cause either poor or no performance monitoring of personnel, resulting in invalid and unreliable results. It could also result in a lack of constructive ideas for improvement and failure to uncover the root causes of poor performance and obstacles in the way of optimal performance (Figure 4).

It is only possible to achieve improved service delivery if the individual employees participate actively in discussions and decision making with their managers pertaining to their work performance.

#### Performance review

Most respondents (69%; *n* = 29) were not familiar with the reasons for having performance reviews during the performance cycle. In the question relating to where the performance review fits in, 22 (56%) respondents answered the question correctly. Twenty-four respondents (62%) did not know how often performance reviews should be conducted and 28 (72%) lacked knowledge of when feedback should be given. It is unclear how the participants, whilst not knowledgeable about the purpose and frequency of monitoring, knew that the performance review was part of the process.

#### Performance assessment

**FIGURE 5:**
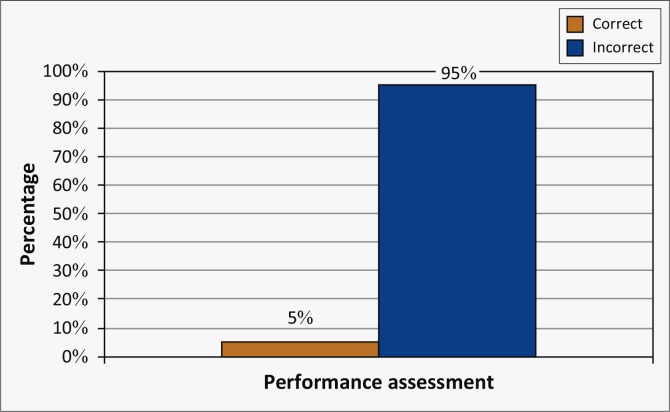
Knowledge regarding the final performance assessment (*N* = 39).

Thirty-seven of the respondents (95%) had limited knowledge regarding the assessment criteria for employee performance and 26 (67%) did not know how to prepare for the final employee performance assessment (Figure 5).

The reason for this was most likely the fact that, because there is such a notably overall lack of knowledge regarding the PMDS, the participants are unable to see the bigger picture. The final performance assessment is crucial in the performance cycle, because management base their decision for promotions, monetary rewards, disciplinary actions and such on the outcome of the final performance assessment (Muthwa 2007:36).

## Limitations of the study

Only 39 professional nurses from two PHC clinics in the Tshwane district of Gauteng province responded to the questionnaire. Therefore, the study size is small and the results can thus not be not be generalised to other PHC clinics in the district or provinces. A follow-up research study with a larger sample size is necessary to determine the knowledge professional nurses poses on the implementation of the PMDS.

The influence of attitude and practices on the implementation of the PMDS was not explored and competence was also not tested for in this study. In the light of the quality health care and government's commitment to provide all South Africans with improved service in the public sector, further research into the attitudes, practices and competence of professional nurses regarding the implementation of the PMDS is suggested. In addition, a qualitative study to obtain valuable insights into professional nurses own perceived knowledge regarding the implementation of the PMDS is suggested, as the PMDS remains a controversial topic.

## Recommendations

The findings of this study highlighteds the important role of professional nurses in the implementation of PMDS and the need to decrease incidents of misunderstanding or knowledge deficits relating to the implementation of the PMDS.

Respondents in this study have limited knowledge about the legislative framework governing the implementation of the PMDS. It therefore seems appropriate for top management to create a supportive and communicative workplace environment to promote taking up ownership for the implementation of the PMDS amongst professional nurses. Respondents attended workshops poorly in this study. It is therefore recommended that training commence under the supervision of experienced professionals by means of continuous training programmes to ensure that professional nurses know the what, the why and the how of such a system.

The performance planning and agreement phase forms not only the cornerstone for the implementation of the PMDS; rather it is an opportunity to understand what good work performance entails and to help employees to take the necessary actions and encouraging them to achieve set goals during the PMDS cycle. The performance planning and agreement at the start of the PMDS cycle should provide an opportunity for the professional nurses and employees to sit down to discuss, clarify and mutually agree on what, why, how and when priority goals and objectives could be achieved. It is thus suggested that a protocol on the implementation of the PMDS should be made readily available in the two PHC clinics, for easy access to information. This could ensure that the performance planning and agreement phase start off correctly.

As PMDS aims to manage the work performance and developed of employees in the public sector, improved quality patient care and service delivery outcomes play an essential role in this effort. It is thus recommended that performance monitoring should be done in accordance with the PMDS requisites, that is, continually. This could ensure that the professional nurses and those reporting to them stay on course and achieve the desired outcomes of safe, cost effective and efficient patient care and service delivery.

A quarterly performance review could enhance smooth organised work practices, fair and supportive leadership for employees to feel confident enough in carrying out task and to implement their own innovative ideas that may promote a sense of togetherness, belonging and meaningfulness in the workplace environment. Respondents in this study clearly indicate inconsistency in carrying out performance reviews. There is no consistency on how often reviews are carried out. It is suggested that performance review should be done on a quarterly basis so that professional nurses could focus on strengthening the positive aspects of the employee work performance and identify areas for improvement. The employees are thus not kept in the dark but know the exact details regarding their level of work performance and ways to correct identified shortcomings within a specified time period.

The final annual performance assessment is an important overall review of the initial goals that was set during the performance planning agreement phase. The outcomes of the performance assessment assist top management in deciding on the future of the organisation and the distribution of incentives, promotions, and reinforcement of improved work performance. For this reason, top management relies solely on the professional nurse to provide honest and authentic assessment results. Preparation for such a meeting is thus deemed important with regard to ensuring that the assessment is carried out both fairly and consistently. It is, therefore, recommended that top management and the human resources department should be involved in coaching, supporting and guiding professional nurses to ensure that they possess the required skills and are competent to conduct a performance assessment session.

## Conclusion

Professional nurses are responsible for and play a notably important role in the implementation of the PMDS in diverse public healthcare settings. This study successfully explored and described knowledge deficiency as a major barrier to PMDS implementation amongst professional nurses working at selected PHC clinics. This knowledge deficiency might directly compromise the quality of service delivery and hinder government's commitment to provide all South Africans with improved service in the public sector. The discussion set out above vindicates the fact that a platform to address the problem needs to be established in the interest of the individual employee.

## References

[CIT0001] Adejoka A.B. & Bayat M.S., 2014, ‘Evaluation of performance management and development systems with balanced scorecard as a performance appraisal tool at Mthatha general hospital – Eastern Cape Province’, *Journal of Research and Development* 1(7), 7–24.

[CIT0002] Bless C., Higson-Smith C. & Kagee A., 2006, *Fundamentals of social research methods: An African perspective*, Juta and Company (Pty) Ltd, Cape Town.

[CIT0003] Brink H.I., van der Walt C. & van Rensburg G., 2006, *Fundamentals of research methodology for health care professionals*, 2nd edn., Juta and Company (Pty) Ltd., Cape Town.

[CIT0004] Department of Local Government and Traditional Affairs, 2007, *Employee performance management and development system [EPMDS] policy guidelines*, KwaZulu-Natal Provincial Government, Pietermaritzburg.

[CIT0005] Hall A., 2005, ‘Defining nursing knowledge’, *Nursing Times* 101(48), 34–37. 16350518

[CIT0006] Hough J., Thompson A.A., Strickland A.J. & Gamble J., 2008, *Crafting and executing strategy: South African edition*, McGraw-Hill Education, Berkshire.

[CIT0007] Kgomotso R.E., 2011, ‘Improving the performance management and development system in the Department of Health and Social Development, Limpopo Province’, Master's thesis, Business Management, University of Limpopo, South Africa.

[CIT0007a] Kirkpatrick D.L., 2006, *Improving employee performance through appraisal and coaching*, 2nd edn., American Management Association, New York.

[CIT0008] Kondrasuk J.N., 2011, ‘So what would an ideal appraisal look like?’, *Journal of Applied Business and Economics* 12(1), 57–71.

[CIT0009] Leedy P.D. & Ormod J.E., 2000, *Practical research: Planning and design*, 7th edn., Pearson Educational International, NJ.

[CIT0010] Letsoalo M.B., 2007, ‘An evaluation of performance management in the public service’, Master's thesis, Human Resource Development and Training, Faculty of Human Resource Management, University of Johannesburg, South Africa.

[CIT0011] LoBiondo-Wood G. & Haber J., 2010, *Nursing research: Methods and critical appraisal for evidence-based practice*, 7th edn., Mosby Elsevier, St Louis, MI.

[CIT0012] Marquis B.L. & Huston C.J., 2009, *Leadership roles and management functions in nursing: Theory and application*, 6th edn., Lippincott Williams and Wilkens, Philadelphia, PA.

[CIT0013] Marriner-Tomey A.M., 2004, *Guide to nursing management and leadership: Middle East and African edition*, 7th edn., Mosby Elsevier, St. Louis, MI.

[CIT0014] Millar R., 2013, ‘Framing quality improvement tools and techniques in healthcare: The case of improvement leaders’ guides’, *Journal of Health Organization and Management* 27(2), 209–224. 10.1108/14777261311321789 23802399

[CIT0015] Mokoka E., Oosthuizen M.J. & Ehlers V.J., 2010, ‘Retaining professional nurses in South Africa: Nurses managers’ perspectives’, *Health SA Gesondheid* 15(1), Art. #484, 9.

[CIT0016] Muller M., Jooste K. & Bezuidenhout M., 2008, *Healthcare service management*, Juta and Company (Pty) Ltd., Cape Town.

[CIT0017] Muthwa S., 2007, *Performance management and development system*, viewed 11 August 2014, from http://www.ecprov.gov.za/page.php?index=39

[CIT0018] Nel P.S., Swanepoel B.J., Kirsten M., Erasmus B.J. & Tsabadi M.J., 2005, *South African employment relations: theory and practice*, 5th edn., Van Schaik Publishers, Pretoria.

[CIT0019] Paile N.J., 2012, ‘Staff perceptions of the implementation of a performance management and development system: Father Smangaliso Mkhatswa case study’, Master's thesis, University of South Africa, South Africa.

[CIT0020] South Africa, 2005, *Nursing Act, 2005* (Act No. 33 of 2005), Government Printers, Pretoria.

[CIT0021] Stavrianopoulos T., 2012, ‘The clinical nurse leader’, *Health Science Journal* 6(3), 392–401.

